# Automated 3D scoring of fluorescence in situ hybridization (FISH) using a confocal whole slide imaging scanner

**DOI:** 10.1186/s42649-021-00053-y

**Published:** 2021-04-09

**Authors:** Ziv Frankenstein, Naohiro Uraoka, Umut Aypar, Ruth Aryeequaye, Mamta Rao, Meera Hameed, Yanming Zhang, Yukako Yagi

**Affiliations:** Department of Pathology, Memorial Sloan Kettering Cancer Center, New York, NY 10065 USA

**Keywords:** Fluorescence in situ hybridization (FISH), Confocal whole slide imaging (WSI) scanner, Automated, Segmentation, Algorithm

## Abstract

Fluorescence in situ hybridization (FISH) is a technique to visualize specific DNA/RNA sequences within the cell nuclei and provide the presence, location and structural integrity of genes on chromosomes. A confocal Whole Slide Imaging (WSI) scanner technology has superior depth resolution compared to wide-field fluorescence imaging. Confocal WSI has the ability to perform serial optical sections with specimen imaging, which is critical for 3D tissue reconstruction for volumetric spatial analysis. The standard clinical manual scoring for FISH is labor-intensive, time-consuming and subjective. Application of multi-gene FISH analysis alongside 3D imaging, significantly increase the level of complexity required for an accurate 3D analysis. Therefore, the purpose of this study is to establish automated 3D FISH scoring for z-stack images from confocal WSI scanner. The algorithm and the application we developed, SHIMARIS PAFQ, successfully employs 3D calculations for clear individual cell nuclei segmentation, gene signals detection and distribution of break-apart probes signal patterns, including standard break-apart, and variant patterns due to truncation, and deletion, etc. The analysis was accurate and precise when compared with ground truth clinical manual counting and scoring reported in ten lymphoma and solid tumors cases. The algorithm and the application we developed, SHIMARIS PAFQ, is objective and more efficient than the conventional procedure. It enables the automated counting of more nuclei, precisely detecting additional abnormal signal variations in nuclei patterns and analyzes gigabyte multi-layer stacking imaging data of tissue samples from patients. Currently, we are developing a deep learning algorithm for automated tumor area detection to be integrated with SHIMARIS PAFQ.

**Supplementary Information:**

The online version contains supplementary material available at 10.1186/s42649-021-00053-y.

## Introduction

Fluorescence in situ hybridization (FISH) is a technique employed fluorescently labeled probes to specifically bind a target genome sequence and it is in research and clinical use (Gozzetti and Le Beau 2000; Kajtar et al. 2006; Tanas et al. 2010; Hu et al. 2014). The technique enables spatial localization of multiple signals within the cell nuclei to provide the presence, location and structural integrity of genes on chromosomes. Applications of FISH assay together with imaging techniques, such as confocal and wide-field fluorescence are commonly in use. However, confocal imaging provides images with higher quality in terms of sharpness, contrast, and noise when compared to wide-field fluorescence imaging (Xiujun Fu et al. 2017). Confocal imaging technology increases optical resolution compared to traditional wide-field fluorescent imaging by means of adding a spatial pinhole placed at the focal plane of the lens to eliminate the out-of-focus light (Wright et al. 1993). However, in wide-field fluorescence the entire specimen of interest is exposed to the light source and the specimen axial dimension should be less than the wave-optical depth to satisfy in-focus condition. This condition limits the portion of the tumor that can be scanned.

Other advantages of confocal fluorescence imaging over wide-field fluorescence imaging are the elimination/reduction of background information from focal plane and lower excitation energy as well as the ability to perform serial optical sections with thick specimen (which is critical for 3D tissue reconstruction). Application of multi-layer Z-stack for 3D tissue reconstruction with FISH assay enables the volumetric spatial visualization of multiple genes signals with different colors within the cell nuclei (Diaspro 2001; Xiujun Fu et al. 2017). Since image acquisition is time-consuming and subjective, whole slide imaging (WSI) technology has been applied to automate the digital image acquisition from glass slide (Brachtel and Yagi 2012; Laurent et al. 2013) with confocal scanner for FISH slide imaging (Xiujun Fu et al. 2017).

Hybridized gene signals on FISH slides have extremely small size and occupy tiny volumes inside the nuclei, with average diameter of several hundred nanometers (Hildenbrand et al. 2005; Xiujun Fu et al. 2017). Microscopy with high magnification objective is required in order to visualize and distinguish these extremely small size signals. The epifluorescence microscopy (wide-field fluorescence microscopy), is commonly used to view FISH slides to count the fluorescent signals for scoring and diagnosis. Most WSI scanners designed for bright field imaging and have 20× or 40× objectives for digital fluorescence imaging with digitize slide optics equivalent to epifluorescence microscopy (Cornish et al. 2012; Laurent et al. 2013). A WSI fluorescence scanner has been used with FISH slide of diffuse large B cells lymphoma cases with break-apart probes to detect MYC rearrangement (Laurent et al. 2013). It shown to be rapid, robust, and highly sensitive. However, these scanners encounter difficulty in capturing the miniscule fluorescence signals from the nuclei when digitizing FISH slide. A confocal WSI scanner recently used with high magnification of 40× objective, producing final image with high pixel resolution of 0.16 μm/pixel, shown to be capable of acquiring each of the fluorescence signals from the FISH slide (Xiujun Fu et al. 2017). Moreover, the extremely small size fluorescent signals which carry the specific genetic information on FISH slides are distributed spatially inside the nuclei volume (Xiujun Fu et al. 2017); and therefore, could not be completely detected by a single-layer scanning method.

The spatial arrangement of genes may reflect normal or rearrangements in chromosomes (Roix et al. 2003; Gue et al. 2005). However, genes visualization by fluorescence probes cannot be interpreted accurately by 2D imaging strategy (due to missing information of the Z-axis) when taking into account the various spatial locations of gene signals inside the cell nuclei. For example, the diagnosis of gene translocation or fusion using FISH break-apart probe requires to measure the spatial distance between gene signals from different channels to determine break-apart or co-localization (fusion) of signals (Alpar et al. 2008; Cornish et al. 2012). This spatial distance between different gene signals is essential for the diagnosis. Here, we use confocal WSI in analysis of FISH signals across Z-stack volume, which allows to precisely localize and detect the spatial distance between gene signals inside the cell nuclei volume. Yet, it is impossible to precisely determine by eye the distance between gene signals within individual cell nuclei. Even though FISH analysis is complicated, clinical cytogeneticists perform counting rely on their experience for gene signal patterns detection and individual nuclei morphology identification. Automated signal quantification is objective and may improve productivity with plentiful information. Therefore, we established automated 3D FISH scoring of z-stack images from confocal WSI scanner. Our algorithm and application, SHIMARIS PAFQ, successfully employs 3D calculations for segmenting clear individual nuclei shapes, gene signals detection, distribution of break-apart probe signal patterns, including standard break-apart, and variant patterns due to truncation, deletion, etc. The analysis was accurate and precise when compared with grand truth clinical manual counting and scoring reported in ten lymphoma and solid tumors cases. Where EWSR1, MYC, BCL2 and BCL6 break apart FISH probes used as a diagnostic guide to determine treatment of lymphoma or solid tumors patients (Sesques and Johnson 2017). The algorithm we developed is objective and more efficient than the current standard clinical procedure. It enables the automated counting of more cell nuclei and detect additional variations in gene signals abnormal patterns within the nuclei than the conventional clinical counting method. As well as accurately retrieve gene signals number and calculate 3D vector lengths between different gene signals for each individual nuclei together with nuclei patterns classification.

## Materials and methods

This study involves human subjects and is therefore approved by the institutional review board of Memorial Sloan Kettering Cancer Center (MSKCC), New York, NY, USA (IRB No. 18–216).

### Tissue sectioning and FISH slides preparation

**Fig. 1:**
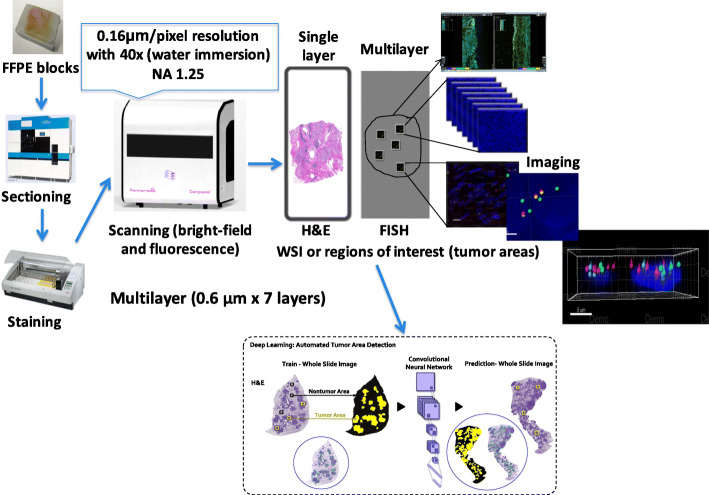
Workflow of tissue sectioning, staining and scanning. Serial sectioning of FFPE tissue blocks was used for H&E or IHC staining in order to characterize the region of interest. H&E and IHC slides were scanned at wide-field mode with 20× water immersion objective at a single layer. ROIs on FISH slides were scanned at confocal mode with multiple layers (*N* = 7 layers at 0.6 μm interval) with 40× water immersion objective and a final image resolution of 0.16 μm/pixel. Three filters were chosen: DAPI (blue), FITC (green) and TRITC (red). Showing in a dashed line frame: we are currently developing a deep learning algorithm for an automated tumor area detection

Information concerning lymphoma and solid tumors patients has been retrieved from MSKCC (Table 1). Tissue samples included in this analysis were formalin-fixed paraffin-embedded (FFPE) blocks. FFPE tissue blocks is suitable for clinical diagnostics due to the preservation procedure were the morphology retain relatively intact (Watters and Bartlett 2002; Kikuchi et al. 2016). Therefore, FISH pretreatment protocol reduces formalin effect to optimize the access of FISH probes to target DNA (Watters and Bartlett 2002). Serial sectioning of FFPE tissue blocks (Fig. 1) was used for Hematoxylin and Eosin (H&E) or immunohistochemistry (IHC) staining in order to characterize the region of interest (ROIs). The AS-410 (Dainippon Seiki Co. LTD., Japan) automated sectioning machine was used. A robotic arm that is guided by a sensor, picks the tissue block to be sectioned. The tissue block is charged positively, cooled, and then humidified before it is sectioned. The positively charged tissue block attaches to a negatively charged carrier tape that transports and deposits it on a glass slide moistened with water droplets (help spread the tissue). The tissue slide heated (to minimize, if not totally remove, wrinkles) and then drying. This H&E or IHC slide was used for tissue orientation to ensure that the correct area in an adjacent slide was selected for FISH scoring. FISH analysis was performed on 4.0 μm section were tissue hybridized with break-apart probe to detect gene rearrangements. FISH slides were prepared for ten patients as follows. Slides were pretreated with buffer solution as well as with hydrochloric acid to solubilizing basic nuclear proteins, improving the accessibility of the DNA. This method extracts the extracellular matrix of proteins to improve accessibility of the probe to the cells and preventing tissue autofluorescence (Watters and Bartlett 2002). Pretreated tissue was digested with buffer and protease for the purpose of breaking of peptide bonds to affect signal quality by allowing access of the FISH probes to the genomic target DNA and reduces autofluorescence generated by intact proteins (Watters and Bartlett 2002; Kikuchi et al. 2016). Protease digestion was terminated by dehydrating slides in an alcohol series and air-dried. FISH probes directly labeled with fluorochromes are commercially available and ready to use in red, green and blue fluorophores. Probes were applied to the tissue slide, cover slipped, sealed and denaturation was conducted and hybridized in a humidified ThermoBrite system. Post hybridization washing preformed at preheated temperature in order to avoid hybrids of low homology. Slides were dehydrated in an alcohol series. Air dried slides were counterstained using Vectashield with 4′,6-diamidino-2-phenylindole (DAPI) medium and cover slipped. Slides were stored at − 20 °C. DAPI targeting the DNA in the cell nucleus with blue fluorophore.

**Table 1:** Dataset for FISH diagnosis of lymphoma and solid tumors patients

Case	Diagnosis	Break-apart probe	Clinical result
1	Diffuse large B-cell lymphoma	BCL6	Negative (−)
2	Follicular lymphoma	BCL2	Negative (−)
3	Ewing’s sarcoma	EWSR1	Positive (+)
4	Diffuse large B-cell lymphoma with plasmacytic differentiation	MYC	Negative (−)
5	In situ follicular neoplasia	BCL2	Positive (+)
6	Focal diffuse large B-cell lymphoma and follicular lymphoma	BCL6	Negative (−)
7	Diffuse large B-cell lymphoma	BCL6	Positive (+)
8	Diffuse large B-cell lymphoma	MYC	Positive (+)
9	Diffuse large B-cell lymphoma	MYC	Negative (−)
10	Diffuse large B-cell lymphoma	MYC	Negative (−)

The FISH dataset includes patients who had been diagnosed with diffuse large B-cell lymphoma (DLBCL), follicular lymphoma, diffuse large B-cell lymphoma (DLBCL) with plasmacytic differentiation, in situ follicular neoplasia and focal diffuse large B-cell lymphoma (DLBCL) with follicular lymphoma. Patients were analyzed with BCL6, BCL2, MYC, EWSR1 and MYC break apart probes as a diagnostic guide to determine treatment. This probes set includes the combinations of the following fluorophores: fluorescein isothiocyanate (FITC) green fluorescent proteins (GFP and EGFP), paired with tetramethylrhodamine isothiocyanate (TRITC) red fluorescent protein (DsRed).

### FISH slides scanning

As shown in Fig. 1, H&E as well as IHC were used for the propose of tumor area detection. IHC interpretation (demonstrates coexpression of markers) was useful when follicular lymphoma/neoplasia were part of the differential diagnosis (Sesques and Johnson 2017): cases 2, 5 and 6. Slides were digitized with the pannoramic confocal scanner (3DHISTECH Ltd., Budapest, Hungary). The optical components of this scanner allow both bright field and fluorescence imaging as well as both wide-field and confocal modes are provided for fluorescence imaging. The scanner supports fully automated scanning and semi-automated scanning. All the calculations in the fully automated scanning to define the focus maps as well as detection of the tissue regions were performed by a control software. However, the semi-automated scanning allows user to define the focus map as well as the tissue regions to be scanned.

H&E and IHC slides were scanned at wide-field mode with 20× water immersion objective at a single layer. Scanned wide-field images were viewed, and several ROIs from each slide were selected within the tumor area of the tissue and reviewed by a pathologist. ROIs on H&E and IHC slides were used in semi-automated mode to define the ROIs on FISH slides. ROIs on FISH slides were scanned at confocal mode with multiple layers for both targeting genes and nuclei visualization. Multi-layer scanning of *N* = 7 layers at 0.6 μm interval were performed. Exposure time of the scans was set based on the signal intensities of each channel with 40× water immersion objective and a final image resolution of 0.16 μm/pixel (has a numerical aperture of 1.2). Three filters were chosen in accordance with their fluorescent excitation and the emission wavelengths of the probes (Supplemental Table 1). The three filters are DAPI, FITC and TRITC (as described above). The source of the scanner excitation light is the Lumencor LED light engine for the highest possible illumination power and PCO edge cooled scientific CMOS camera combining high sensitivity and low noise.

### Image evaluation and analysis

WSI images were visually assessed and annotated in CaseViewer provided by 3DHISTECH. The tumor areas were semi-automatically detected on FISH slides as described above. The annotated regions were exported into tiled TIFFs (representation of each layer of the multi-layer scanning). The exported tiled TIFFs were imported into our algorithm for clear individual cell nuclei segmentation and gene signals detection, quantification, co-localization and 3D analysis as described below. Analysis of gene signals corresponding to individual cell nuclei were performed using our algorithm. The accuracy of the analysis was compared with manual investigation as assessed clinically by pathologist and cytogeneticists, where overlapping red and green or fused yellow signal represents co-localization, and separate red and green signals indicate break-apart. Unlike the clinical manual investigation, our new algorithm calculates the 3D vector length between different channels. Thus, the diameters of gene signals spots both in FITC and TRITC channels were set as 0.6 μm and the cut-off 3D distance to define break-apart gene signals was set to 1.2 μm (twice or more than the gene signal spot diameter). The negative diagnosis of patient relies on the mentioned relationship of gene signals inside each individual nuclei, where 10% or less in counted individual nuclei shows abnormal signal patterns.

### Algorithm description for 3D scoring of FISH using a confocal WSI scanner

**Fig. 2:**
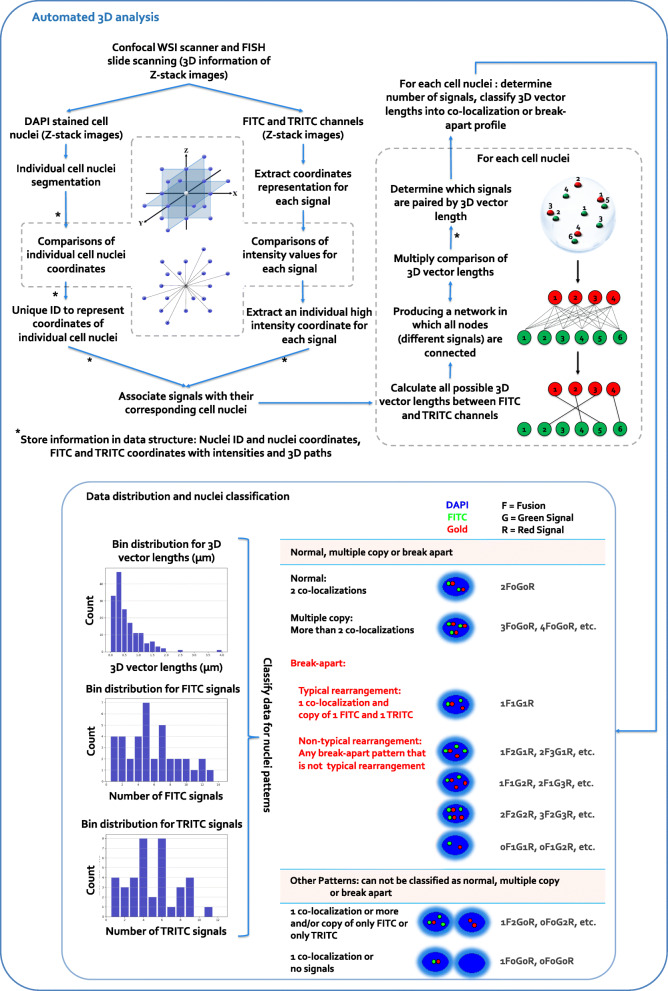
Algorithm description for 3D scoring of FISH using confocal WSI scanner. Steps are described for 3D calculations for clear individual cell nuclei segmentation, gene signals detection and distribution of break-apart probes signal patterns, including standard break-apart, and variant patterns due to truncation, and deletion, etc.

**Fig. 3:**
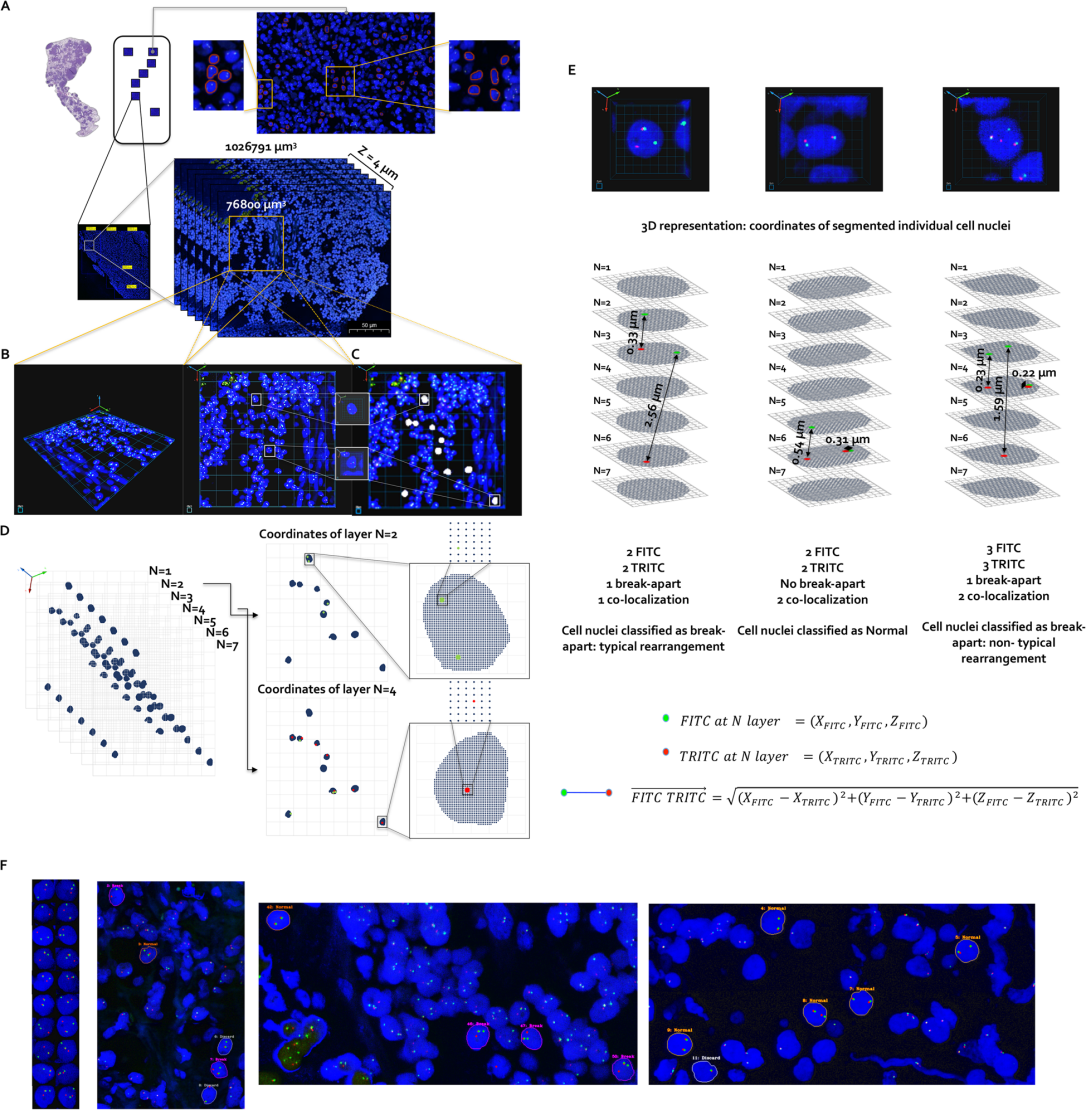
Segmentation and coordinates representation of signals to determine 3D co-localization, break-apart and other variations in individual cell nuclei patterns. **a** Volume and segmentation representation. Showing Z-stack images scanned with 7-layer and 0.6 μm interval at the same area. Blue is DAPI channel for stained nuclei, green is FITC channel, and red is TRITC channel. **b** 3D representation of selected volume from panel A. **c** Segmentation of clear individual cell nuclei (shown in gray) found at the volume. **d** Coordinates representation of segmented signals from the 7-layers Z-stack. **e** 3D vector length calculation using the X, Y and Z coordinates extracted from the 7-layers Z-stack (to determine co-localization and break-apart between FITC and TRITC signals) and classification of cell nuclei pattern. **f** Collection of segmented individual cell nuclei to show variations in signals patterns (normal in orange frame, break in purple frame and other patterns in gray frame)

The algorithm is described in Fig. 2 and illustrated in Fig. 3. 3D information of Z-stack images were exported into tiled TIFFs data. Exported tiled TIFFs were imported into our algorithm. We employed Gaussian filter to reduce noise. This is a non-linear low-pass filter that removes high-frequency components. Gaussian function is given as:

$$ f(x)=\raisebox{1ex}{$1$}\!\left/ \!\raisebox{-1ex}{$\sigma \sqrt{2\pi }$}\right.\mathit{\exp}\left(\raisebox{1ex}{${\left(x-\mu \right)}^2$}\!\left/ \!\raisebox{-1ex}{$2{\sigma}^2$}\right.\right) $$

where μ is mean and σ is variance.

In addition, we operated morphological opening and closing transformation for noise removing, isolation of individual elements and joining disparate elements as well as finding of intensity bumps or holes. Opening obtained by erosion followed by a dilation which results in removing small objects on the foreground:

$$ dst= open\ \left( src, element\right)= dilate\ \left( erode\ \left( src, element\right)\right) $$

Closing is reverse of opening and obtained by dilation followed by erosion. It is useful in closing small holes inside the objects:

$$ dst= close\ \left( src, element\right)= erode\ \left( dilate\ \left( src, element\right)\right) $$

A template matching technique was employed to segment DAPI stained clear individual cell nuclei. We used the technique to find the statistically significant match between an individual nuclei templates and the target image (Gihan Kuruppu and Pinidiyaarachchi 2013). The size of the source image I is W × H where W and H representing the width and height, respectively. The source image I was compared with the overlapped patches of the template image T (with width “w” and height “h”). The template moves one pixel in the horizontal or vertical direction on the image to be tested and performs a comparison calculation. All possible locations to be matched with the template are stored in a resultant matrix R given by (W – w + 1) × (H – h + 1) which stores the coefficient value for each matched location in pixel. We tested different approaches for nuclei segmentation, some based on pixel by pixel intensity differences to calculate the summation of squared (Ourselin et al. 2001; Di Stefano and Mattoccia 2003). Other approaches are more complex as they involve numerous multiplication, division and square root operations (Wei and Lai 2008). The different approaches described bellow, where x and y are the source pixel position and u and v are variable, shift component along x-direction and y-direction respectively.

Square difference matching is defined as:

$$ {R}_{SD}\left(x,y\right)=\sum \limits_{u,v}{\left(T\left(u,v\right)-I\left(x+u,y+v\right)\right)}^2 $$

Normalized square difference is defined as:

$$ {R}_{NSD}\left(x,y\right)=\raisebox{1ex}{${\sum}_{u,v}{\left(T\left(u,v\right)-I\left(x+u,y+v\right)\right)}^2$}\!\left/ \!\raisebox{-1ex}{$\sqrt{\sum_{u,v}T{\left(u,v\right)}^2\cdot {\sum}_{u,v}I{\left(x+u,y+v\right)}^2}$}\right. $$

Cross correlation matching is defined as:

$$ {R}_C\left(x,y\right)=\sum \limits_{u,v}\left(T\left(u,v\right)\cdot I\left(x+u,y+v\right)\right) $$

Normalized cross correlation matching is defined as:

$$ {R}_{NC}\left(x,y\right)=\raisebox{1ex}{${\sum}_{u,v}\left(T\left(u,v\right)\cdot I\left(x+u,y+v\right)\right)$}\!\left/ \!\raisebox{-1ex}{$\sqrt{\sum_{u,v}T{\left(u,v\right)}^2\cdot {\sum}_{u,v}I{\left(x+u,y+v\right)}^2}$}\right. $$

Correlation coefficient matching is defined as:

$$ {R}_{CC}\left(x,y\right)=\sum \limits_{u,v}\left(T\hbox{'}\left(u,v\right)\cdot I\hbox{'}\left(x+u,y+v\right)\right) $$

where

$$ T\hbox{'}\left(u,v\right)=T\left(u,v\right)-\raisebox{1ex}{$1$}\!\left/ \!\raisebox{-1ex}{$\left(w\cdot h\right)$}\right.\cdot \sum \limits_{u,v}T\left(u,v\right) $$

$$ I\hbox{'}\left(x+u,y+v\right)=I\left(x+u,y+v\right)-\raisebox{1ex}{$1$}\!\left/ \!\raisebox{-1ex}{$\left(w\cdot h\right)$}\right.\cdot \sum \limits_{u,v}I\left(x+u,y+v\right) $$

Normalized correlation coefficient matching is defined as:

$$ {R}_{NcC}\left(x,y\right)=\raisebox{1ex}{${\sum}_{u,v}\left(T\left(u,v\right)\cdot I\left(x+u,y+v\right)\right)$}\!\left/ \!\raisebox{-1ex}{$\sqrt{\sum_{u,v}T{\left(u,v\right)}^2\cdot {\sum}_{u,v}I{\left(x+u,y+v\right)}^2}$}\right. $$

We found no significant differences between the six template matching algorithms. Normalized correlation coefficient and correlation coefficient methods are almost perfectly matching with the ground truth segmentation. Normalized square difference, square difference, normalized cross correlation and cross correlation method have minor variations compared to ground truth segmentation. Also, there is no significant difference between the six template matching algorithms on processing time. The normalized correlation coefficient method performs slightly better in terms of processing time compared to the correlation coefficient method. Therefore, we selected the normalized correlation coefficient matching approach for clear individual nuclei shapes segmentation (Fig. 3). In order to distinguish between the different segmented nuclei by different unique identifier we used connected components of a hypergraph method. A connected component of a hypergraph is defined as any maximal set of vertices which are pairwise connected by a non-trivial path. A vertex of a hypergraph considered to be an isolated vertex if it is not contained in any edge of the hypergraph. If a vertex of the hypergraph is contained in an edge of a particular size, then it is not considered isolated from a specific description of a nuclei. Nuclei coordinates were extracted and compared to assure 3D representation of individual cell nuclei across the layers. The FITC and TRITC channels located inside each individual nuclei were converted into coordinates representation and the high intensity coordinate for each 3D gene signal was extracted by comparing coordinate’s intensities. Figure 2 illustrates the 3D coordinates comparisons we employed. Any selected coordinate consisting of up to 24 neighboring (coordinates) were compared with the selected one for our data representation decisions.

Co-localization and break-apart gene signals where calculated using network representation of 3D vector lengths (between different gene signals) followed by multiply comparisons of the 3D vector lengths (Fig. 2). The network is a weighted network, with each edge assigned a score, representing the 3D vector lengths of the physical interaction between the two signals. Higher score indicates larger 3D distance for the interaction. The distance between two signals with a link in the network is defined as $\overrightarrow{FITC\ TRITC}$, so that smaller $\overrightarrow{FITC\ TRITC}$ would correspond to shorter 3D distance for the interaction. We calculated all possible pairs of different signals directly linked in the network. A key step is the calculations of the network distances. Given the network distance between FITC signal and TRITC signal, we then computed their 3D distance as:

$$ \overrightarrow{ FITC\ TRITC}=\sqrt{{\left({X}_{FITC}-{X}_{TRITC}\right)}^2+{\left({Y}_{FITC}-{Y}_{TRITC}\right)}^2+{\left({Z}_{FITC}-{Z}_{TRITC}\right)}^2} $$

where (*X*_*FITC*_, *Y*_*FITC*_, *Z*_*FITC*_) are the coordinates representation of FITC signal at layer N_FITC_ and (*X*_*TRITC*_, *Y*_*TRITC*_, *Z*_*TRITC*_) are the coordinates representation of TRITC signal at layer N_TRITC_. Given a list of all possible 3D vector lengths for each individual cell nuclei, we made the selection for interacting signals based on ranking distances with non-repetitive signals (across selected paths). For a given nuclei, the 3D vector lengths can be compared with each other, but not across different nuclei. To make them comparable across each individual nuclei, we first sorted all 3D distances by ranking in increasing order. Then, the top rank (sorter 3D distance) was selected as an interaction, where we removed the lower weights (longer 3D distances) calculated based on at least one same signal as found in the top rank. The sorting process continued without the top rank at each iteration of which was saved as 3D interaction and until list of all possible 3D vector lengths was completed. Distribution of gene signals number and the calculated 3D vector lengths were output together with nuclei patterns classification. Individual nuclei patterns were classified based on number of co-localization and break-apart cases as well as copy number of signals (non interacting signals).

### Application description for 3D scoring of FISH using a confocal WSI scanner

**Fig. 4:**
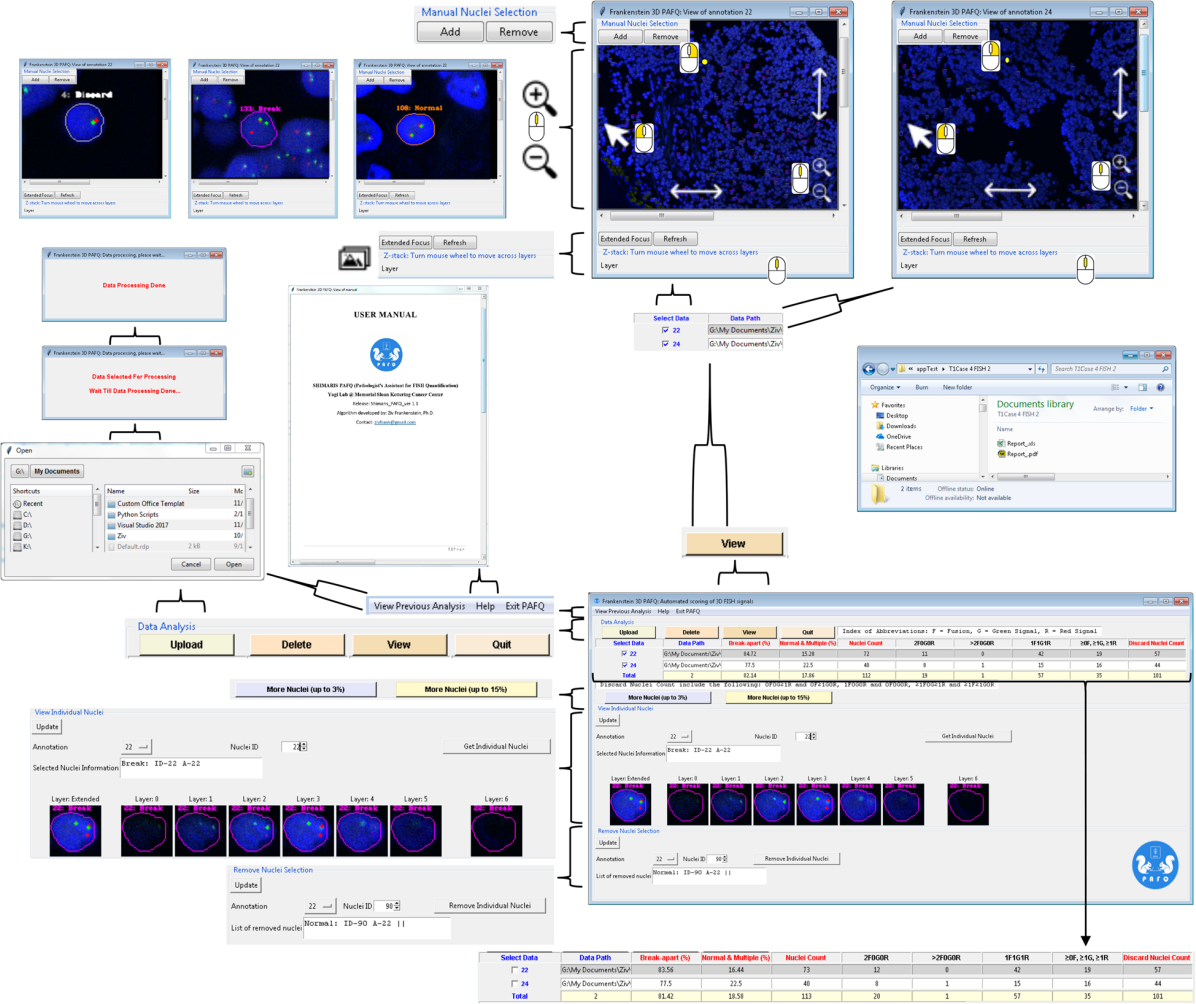
Application description for 3D scoring of FISH using confocal WSI scanner. Flowchart showing application functions, such as 3D data uploading, 3D data deletion, viewing of counting and scoring results, selecting and removing an individual nuclei from the calculations, z-stack image zooms and translations view, export and view of clinical report, quit the software, as well as assistance through manual view

The application we developed, SHIMARIS PAFQ (Fig. 4), includes several functions, such as 3D data uploading, 3D data deletion, viewing of counting and scoring results (break-apart ratio, normal and multiple ratio, total number of counted nuclei, number of counted nuclei for each pattern and number of discard nuclei), selecting and removing an individual nuclei from the calculations, z-stack image zooms and translations view (with the option to move across layers), export and view of clinical report, quit the software, as well as assistance through manual view. This approach was successful for analyzing gigabyte multi-layer stacking imaging data of tissue samples. This application allows users to analyze the data by pressing optional buttons. In addition, the application responds to a successful function through message, and if the application detects failure or an error, it provides useful messages that assist users to make the necessary correction. The application provides a friendly user interfaces to analyze the data.

## Results

**Fig. 5:**
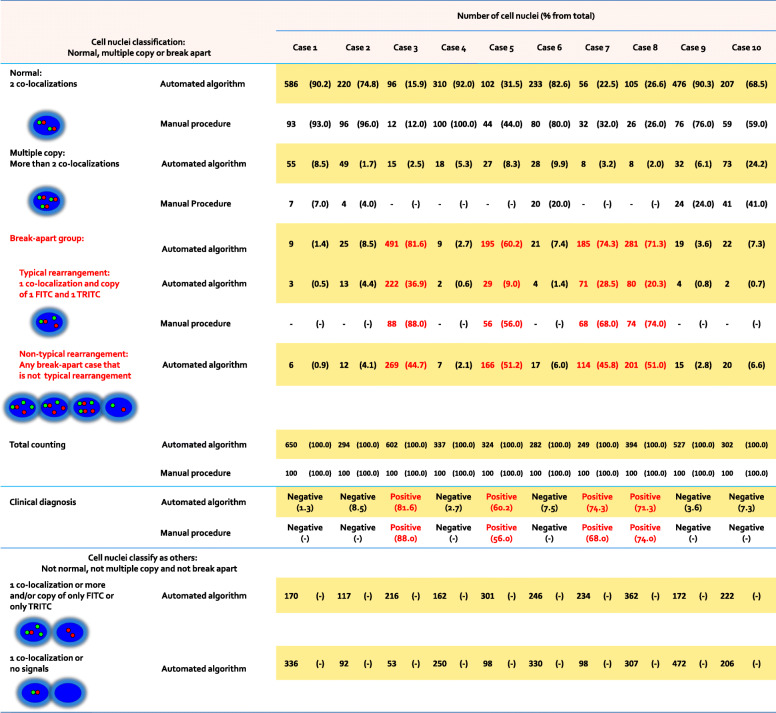
3D FISH counting and scoring of individual cell nuclei with FITC (green) and TRITC (red) channels. Nuclei patterns are illustrated (left column: normal, multiple copy, break apart or others) and results are shown for the automatic and the clinical manual procedure (nuclei counting with percentage from total). Outcome of positive or negative diagnosis for the lymphoma and solid tumors patients are shown as well

FISH diagnosis with two or more different fluorescence probes can be applied to one sample (Li et al. 2014, 2015) and relies on the number or the local relationship of gene signals within an individual cell nuclei. The current clinical analysis to interpret FISH signals by manually counting and scoring of individual cell nuclei under fluorescence microscope is time-consuming and subjective. Clinical manual analysis is especially complicated when applying multi-gene FISH assay together with confocal WSI scanning (Z-stack information). Due to the high level of complexity required for the 3D analysis we have developed an algorithm for quantification and co-localization analysis of confocal WSI scanned FISH images. The time to analyze volume of the entire tumor is 3.4 min. The algorithm allows 3D analysis of FISH Z-stack images with 2 distinct channels or more. Number of clear individual cell nuclei and number of gene signals in each channel were quantified automatically, as well as the 3D vector lengths between the different channels. Distribution of data was output together with individual nuclei patterns classification. The algorithm was validated against ten clinical cases that were analyzed manually by pathologist and cytogeneticists (Table 1). Figure 5 shows that the automated analysis is significantly correlated with the validation procedures in detecting the expected outcome of positive or negative diagnosis for all the lymphoma and solid tumors patients. Moreover, the algorithm produces extensively more information that the clinical manual scoring. The algorithm performed the fastest calculations in a significantly short time (3.4 min for entire tumor area) than the procedure used for validation. The clinical manual counting is of 100 individual nuclei for each patient, while the automatic procedure was several times more of individual nuclei for each patient. The automatic diagnosis procedure and the procedure used for validation are significantly correlated in detecting nuclei pattern for rearrangement with one co-localization and an additional one FITC and one TRITC gene signals (typical rearmament as shown in Fig. 5). However, the automatic procedure can detect more variations in nuclei patterns for rearrangement than the clinical manual scoring. Such as one co-localization and an additional two or more FITC gene signals with additional one TRITC gene signal or more than one co-localization and an additional one FITC gene signal with additional two or more TRITC gene signals (non-typical rearmament as shown in Fig. 5). Non-typical rearmament patterns show features that are different from the typical rearmament pattern. Non-typical rearmament patterns seem to be important to determine diagnosis, since in most of the cases the fraction of nuclei counting in that group is significantly higher than the typical rearmament nuclei counting.

Concerning the normal and multiple copy nuclei patterns, automatic diagnosis procedure and the procedures used for validation are significantly correlated. Individual nuclei with normal pattern is characterized with only 2 co-localizations, while multiple copy nuclei pattern is characterized with more than 2 co-localizations (where all signals are infusion). In most cases, normal pattern counting is significantly higher than the multiple copy pattern. Also, when compared with the clinical manual scoring, the automatic procedure can detect other nuclei patterns that cannot be classified as normal, multiple copy or break apart. Other nuclei patterns show features such as one or more co-localization and an additional two or more FITC gene signals with no TRITC gene signals. The other nuclei patterns seem to be significant, since many nuclei counted to be in that group compared with the normal, multiple copy and break apart incidences. While both the automatic procedure and the procedures used for validation determined the same outcome of positive or negative diagnosis, we found specific differences in counting. This is due to differences in the techniques used for counting and scoring.

## Discussion

As described above, the 3D analysis for the organization and alteration of chromosomes and genes by FISH using a confocal WSI scanner is significant. The individual genes visualized by fluorescence probes localized in various locations within the cell nuclei, cannot be interpreted accurately by 2D imaging strategy, such as determine rearrangements, while the relative 3D position of genes permits precise localization. Moreover, the current clinical manual FISH counting and scoring under fluorescence microscope is time-consuming and subjective. Application of multi-gene FISH analysis (with two or more different fluorescence probes in one sample (Li et al. 2014, 2015) together with 3D imaging, significantly increase the level of complexity required for an accurate 3D analysis. Hence, we developed an automated algorithm and application, SHIMARIS PAFQ, for 3D quantification, co-localization and abnormal signal patterns analysis of confocal WSI scanned FISH z-stack images with 2 distinct channels or more. The algorithm performs 3D automatic analysis of FISH Z-stack images to count the number of clear individual cell nuclei, the number of gene signals and the 3D vector length between the different channels in each cell nuclei. Distribution of signals and the 3D vector lengths were output together with individual cell nuclei patterns classification. Automatic calculations was conducted in a significantly shorter time (3.4 min for the entire tumor area) than the procedure used for validation, clinical manual scoring. For all lymphoma and solid tumors patients, the algorithm detected the same outcome of positive or negative diagnosis as detected using the validation procedure. While nuclei patterns counting classified as normal is significantly higher than the multiple copy pattern. The multiple copy pattern requires further investigation concerning the number of co-localizations found within each cell nuclei. Yet, the algorithm counted several times more of individual cell nuclei for each patient than the clinical manual counting. Since the algorithm produced a relatively larger amount of information than the clinical manual procedure, there are specific differences in counting and the patterns detected. The algorithm detected more variations in nuclei patterns classified as rearrangement, while the combination between gene signals is open to any break apart feature (Fig. 5, non-typical rearrangement). For example, more than one co-localization and an additional several FITC gene signal with additional of several TRITC gene signals. That variations in the break apart features seem to be important, since in most cases the fraction of that group is significant when a positive diagnosis is determined. Also, the algorithm detected other nuclei patterns that cannot be classified as normal, multiple copy or break apart and many nuclei were counted to be in this group which makes it significant. For example, only one co-localization and no additional FITC gene signals or TRITC gene signals. The variations found automatically in nuclei patterns requires further investigation that may improve diagnosis.

We are currently developing a deep learning algorithm for automated tumor area detection to be integrated with SHIMARIS PAFQ (Fig. 1). The deep learning algorithm is trained to identify tumor region compared with that of the nontumor area.

## Conclusion

We established automated 3D FISH scoring (multi-gene) for z-stack images from confocal WSI scanner. The standard clinical manual scoring for FISH is labor-intensive, time-consuming and subjective. Application of multi-gene FISH analysis alongside 3D imaging, significantly increase the level of complexity required for an accurate 3D analysis. Therefore, the procedure we developed successfully employs 3D calculations for individual cell nuclei segmentation, gene signals detection and distribution of break-apart probes signal patterns, including standard break-apart, and variant patterns due to truncation, and deletion, etc. The procedure enables the automated counting of more nuclei, precisely detecting additional abnormal signal variations in nuclei patterns than the conventional clinical counting method. As well as analyzes gigabyte multi-layer stacking imaging data of tissue samples from patients.

## Supplementary Information

**Additional file 1: Supplemental Table 1.** Fluorescent excitation and the emission wavelengths of the probes. DAPI, SpGold and FITC were used in this study.

## Data Availability

Please contact author for data requests.
