# Supplementary material for: Automated 3D scoring of fluorescence in situ hybridization (FISH) using a confocal whole slide imaging scanner
Source: Appl Microsc. 2021 Apr 9;51:4. doi: 10.1186/s42649-021-00053-y (PMC8035347; doi:10.1186/s42649-021-00053-y)
Supplement: Supplementary file 1 — Additional file 1: Supplemental Table 1. Fluorescent excitation and the emission wavelengths of the probes. DAPI, SpGold and FITC were used in this study. [file 42649_2021_53_MOESM1_ESM.docx]

## Supplemental table 1. Fluorescent excitation and the emission wavelengths of the probes. DAPI, SpGold and FITC were used in this study.
